# Intravascular Lithotripsy Balloon Entrapped in Right Coronary Artery Requiring Surgical Bailout

**DOI:** 10.1016/j.jaccas.2026.110448

**Published:** 2026-09-24

**Authors:** Pranev Sharma, Helen Routledge, Jasper Trevelyan

**Affiliations:** Worcestershire Acute Hospitals NHS Trust, Worcester, United Kingdom

**Keywords:** acute coronary syndrome, complication, percutaneous coronary intervention

## Abstract

**Background:**

A clinical case of an entrapped intravascular lithotripsy (IVL) balloon, requiring surgical bailout.

**Case Summary:**

A 72-year-old gentleman presented with a non–ST-segment elevation myocardial infarction. A severe mid right coronary artery calcific lesion was the culprit. We attempted to treat this with percutaneous coronary intervention using IVL. After completion of IVL therapy, the balloon was unable to be freed. All strategies to free the balloon were unsuccessful (a 3-wire wrap, inflating a balloon in parallel and snaring). The patient was sent for emergency coronary artery bypass graft surgery. The IVL balloon could not be freed by the surgeon and so was left cut at the ostium.

**Discussion:**

To our knowledge, this is the only such case report highlighting an entrapped IVL balloon, requiring surgical bailout.

**Take-Home Message:**

Our learning from this case is that when using IVL, resistance to crossing should prompt reassessment of calcium modification strategy rather than continued forceful advancement.

## Visual Summary

**Figure:**
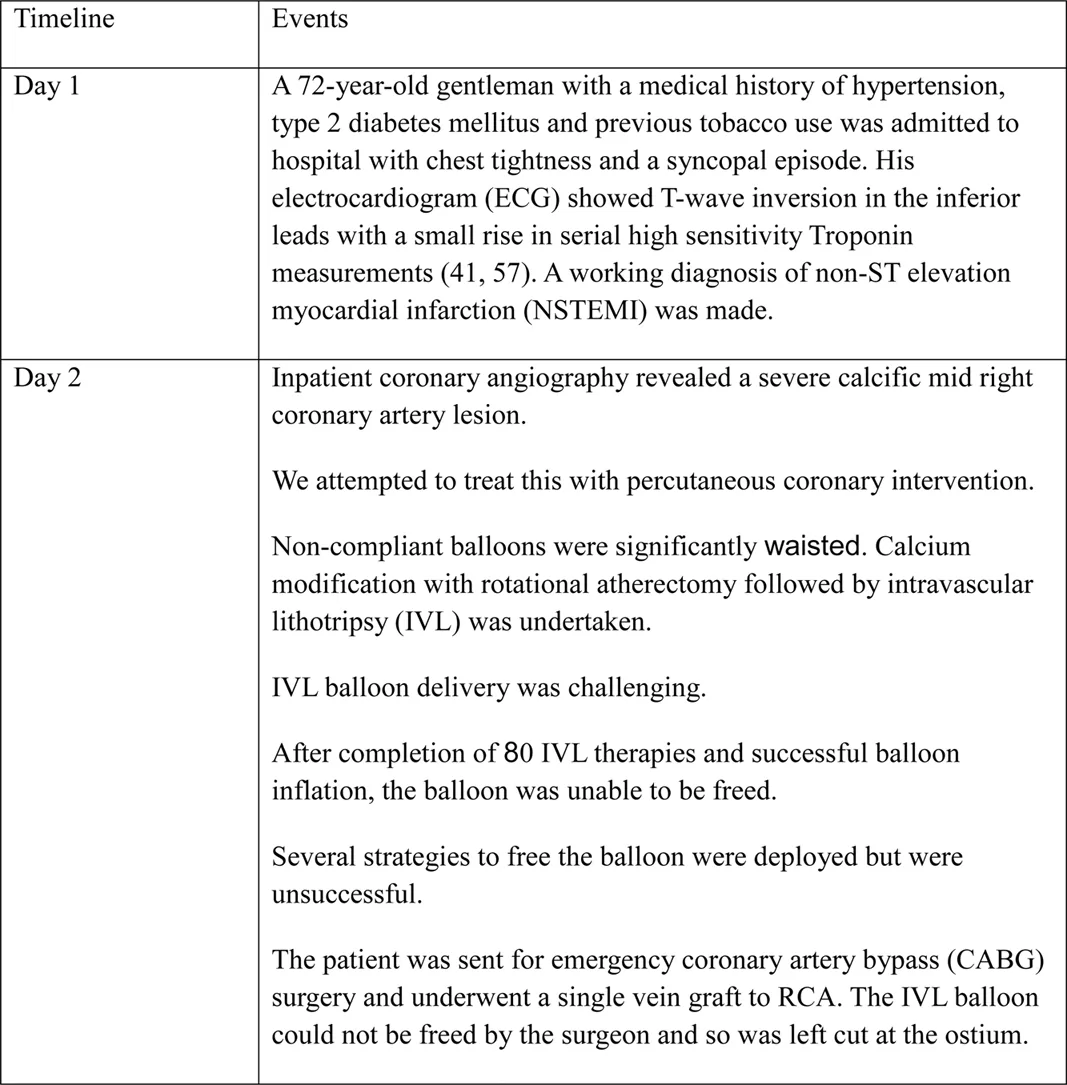

## History of Presentation

A 72-year-old gentleman was admitted to the hospital with chest tightness and a syncopal episode. Physical examination was unremarkable.

## Past Medical History

His medical history included hypertension, type 2 diabetes mellitus, and previous tobacco use.

## Differential Diagnosis

A working diagnosis of non–ST-segment elevation myocardial infarction was made.

## Investigations

His electrocardiogram showed T-wave inversion in the inferior leads with a small rise in serial high-sensitivity Troponin measurements (41, 57).

Echocardiography showed normal biventricular function, no regional wall motion abnormalities, and no significant valvular disease.

## Management

Inpatient coronary angiography was undertaken via the right radial artery. This revealed moderate mid left anterior descending disease, mild left circumflex disease, and severe calcific mid right coronary artery (RCA) disease (Video 1).

The RCA lesion was deemed the culprit and was treated. A Judkins right 4 guiding catheter and a workhorse floppy wire were used. A 2.5 × 15-mm noncompliant balloon was significantly waisted, with little yielding of the lesion (Figure 1).

**Figure 1:**
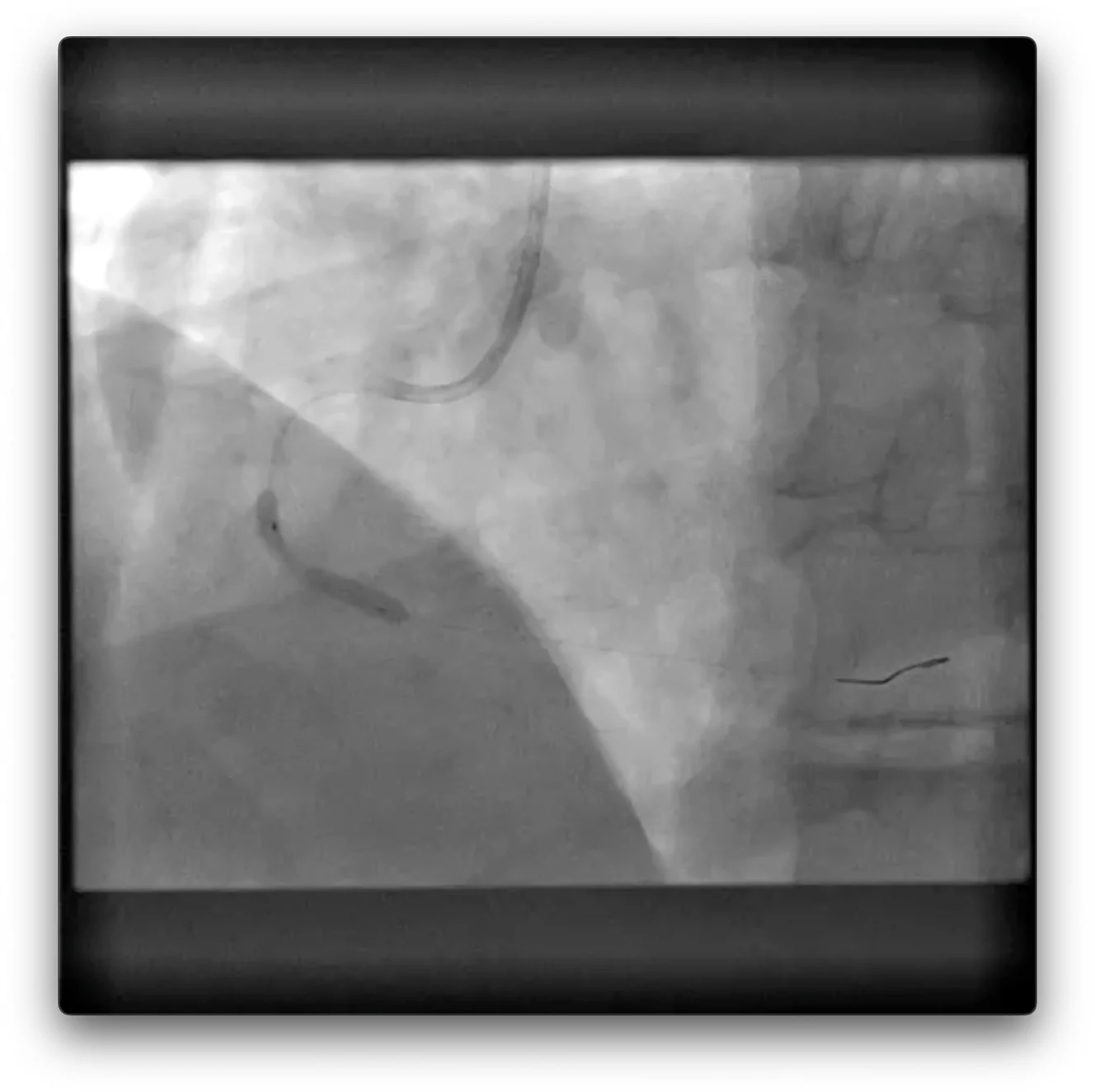
A 2.5 × 15-mm Noncompliant Balloon Was Significantly Waisted, With Little Yielding of the Lesion

Because of challenging delivery of balloons, the guiding catheter was changed to an Amplatz left 0.75 guiding catheter with a 6-F guide catheter extension to assist. Despite this, balloon delivery remained difficult.

Calcium modification in the form of rotational atherectomy was then undertaken with a 1.25-mm burr (Video 2) with evidence of calcium debulking (Video 3). A 3.0-mm noncompliant balloon was then delivered with the aid of a buddy wire and inflated to high pressure but a significant waist remained with insufficient lesion yield (Video 4).

Further calcium modification was deemed necessary with 3.0 × 12-mm intravascular lithotripsy (IVL, Shockwave Medical, Johnson & Johnson, first generation). Despite the use of a guide extension, delivery was challenging, requiring significant “push” force to cross the lesion. A total of 80 IVL therapies were delivered to the lesion, and the IVL balloon inflated without a waist (Figure 2).

**Figure 2:**
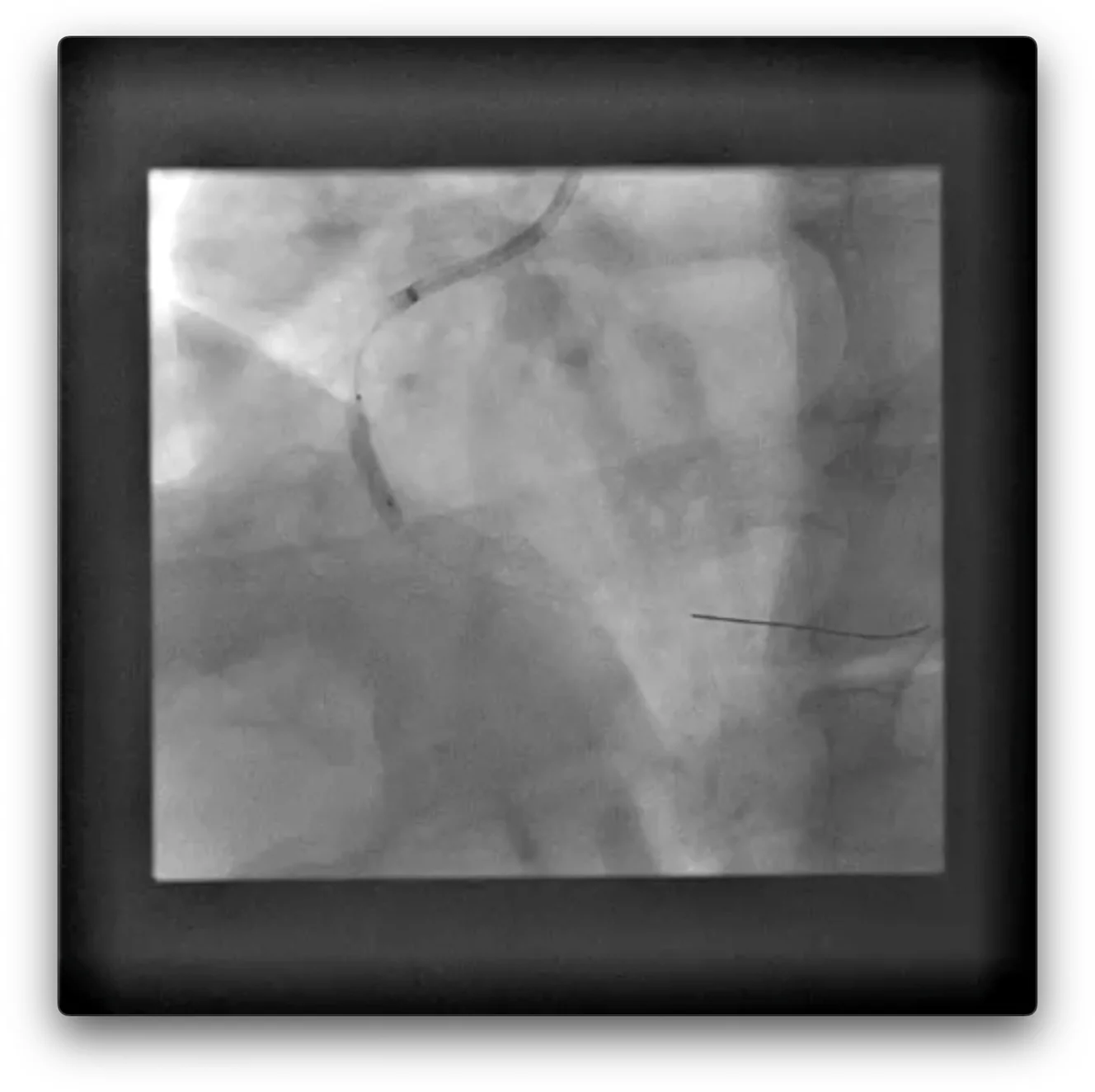
A Total of 80 IVL Therapies Were Delivered to the Lesion, and the IVL Balloon Was Seen to Inflate Without Waisting

On attempting to remove the IVL balloon, however, it would not come back. We tried to pass a wire in parallel but were unable to do so (Video 5). We then obtained arterial access via the left radial artery, used a second guiding catheter, and managed to pass a wire past the entrapped IVL balloon. A 1.25-mm balloon was advanced on this wire and gently inflated in parallel to attempt to free the IVL balloon (Video 6). This maneuver was unsuccessful, and in attempting to pull the IVL balloon back, the shaft of the balloon snapped at the level of the right subclavian artery.

We then attempted to deliver a tulip snare and goose neck snare to the broken shaft at the level of the subclavian (Video 7). This maneuver was also unsuccessful and resulted in a subclavian dissection (Video 8).

At this point, we decided to stop, and the patient was referred for emergency surgery. He received a single vein graft to the RCA. The surgeon was unable to free the IVL balloon with direct pulling, so it was cut at the ostium and left in situ. The subclavian dissection was conservatively managed because antegrade perfusion was intact.

The patient made an unremarkable postoperative recovery and was discharged home 4 days later on lifelong aspirin 75 mg daily. He remains well under clinic follow-up. Routine imaging surveillance has not been deemed necessary for clinical care. General preventive measures for endocarditis are recommended, as per European Society of Cardiology guidance.^1^

## Discussion and Conclusions

Entrapped or broken angioplasty balloon catheters have been described in the previous literature.^2^, ^3^, ^4^ To our knowledge, this is the only described report of an entrapped IVL balloon requiring surgical bailout. The purported mechanism of this “stuck” balloon may relate to the metallic emitters embedding into a disrupted calcium nodule.

**Take-Home Messages**

• Our learning from this case is that when using intravascular lithotripsy, resistance to crossing should prompt reassessment of calcium modification strategy rather than continued forceful advancement.
• If unable to cross a lesion easily with this balloon, one should conclude that further calcium debulking with rotational or orbital atherectomy is required.
• Intravascular imaging, had it been able to cross the lesion, could have allowed us a better understanding of the nature of the calcium in the lesion.

## Funding Support and Author Disclosures

The patient’s treatment was funded by the National Health Service of England. The authors have reported that they have no relationships relevant to the contents of this paper to disclose.

## Appendix

Video 1 Moderate Mid Left Anterior Descending Disease, Mild Left Circumflex Disease, and Severe Calcific Mid Right Coronary Artery Disease

Video 2 Calcium Modification in the Form of Rotational Atherectomy Was Then Undertaken With 1.25-mm Burr

Video 3 Calcium Debulking After Rotational Atherectomy

Video 4 Significant Waisting Remained With Insufficient Lesion Yielding

Video 5 Attempt to Pass a Wire in Parallel But Were Unable to Do So

Video 6 A 1.25-mm Balloon Was Advanced on this Wire and Gently Inflated in Parallel to Attempt to Free the IVL Balloon

Video 7 Attempted to Deliver a Tulip Snare and Goose Neck Snare to the Broken Shaft at the Level of the Subclavian

Video 8 Subclavian Dissection
